# Lifestyle factors and the metabolic syndrome in Schizophrenia: a cross-sectional study

**DOI:** 10.1186/s12991-017-0134-6

**Published:** 2017-02-15

**Authors:** Adrian Heald, John Pendlebury, Simon Anderson, Vinesh Narayan, Mark Guy, Martin Gibson, Peter Haddad, Mark Livingston

**Affiliations:** 1 Department of Medicine, Leighton Hospital, Crewe, CW1 4QJ Cheshire UK; 2 The School of Medicine and Manchester Academic Health Sciences Centre, University of Manchester, Manchester, M13 9PT UK; 3 Greater Manchester West Mental Health NHS Foundation Trust, Greater Manchester, UK; 4 Institute of Cardiovascular Sciences, University of Manchester, Manchester, UK; 5 Department of Clinical Biochemistry, Salford Royal Hospital, Salford, M6 8HD UK; 6 Department of Blood Sciences, Walsall Manor Hospital, Walsall, WS2 9PS UK

**Keywords:** Diet, Lifestyle, Schizophrenia, Metabolic syndrome

## Abstract

**Background:**

Cardiometabolic disease is more common in patients with schizophrenia than the general population.

**Aim:**

The purpose of the study was to assess lifestyle factors, including diet and exercise, in patients with schizophrenia and estimate the prevalence of metabolic syndrome.

**Methods:**

This is a cross-sectional study of a representative group of outpatients with schizophrenia in Salford, UK. An interview supplemented by questionnaires was used to assess diet, physical activity, and cigarette and alcohol use. Likert scales assessed subjects’ views of diet and activity. A physical examination and relevant blood tests were conducted.

**Results:**

Thirty-seven people were included in the study. 92% of men had central adiposity, as did 91.7% of women (International Diabetes Federation Definition). The mean age was 46.2 years and mean illness duration was 11.6 years. 67.6% fulfilled criteria for the metabolic syndrome. The mean number of fruit and vegetable portions per day was 2.8 ± 1.8. Over a third did not eat any fruit in a typical week. 42% reported doing no vigorous activity in a typical week. 64.9% smoked and in many cigarette use was heavy. The Likert scale showed that a high proportion of patients had insight into their unhealthy lifestyles.

**Conclusions:**

Within this sample, there was a high prevalence of poor diet, smoking and inadequate exercise. Many did not follow national recommendations for dietary intake of fruit and vegetables and daily exercise. These factors probably contribute to the high prevalence of metabolic syndrome. Many had insight into their unhealthy lifestyles. Thus, there is potential for interventions to improve lifestyle factors and reduce the risk of cardiometabolic disease.

**Electronic supplementary material:**

The online version of this article (doi:10.1186/s12991-017-0134-6) contains supplementary material, which is available to authorized users.

## Background

People with schizophrenia suffer from increased morbidity and mortality compared with the general population, having a life expectancy that is approximately 20% shorter [1]. The excess mortality is largely due to cardiovascular disease (CVD). Furthermore, people with schizophrenia and other severe and enduring mental illnesses (SMI) are twice as likely to die from CVD compared with those in the general population [2–4], and the excess mortality is higher in younger individuals. Known risk factors for CVD include smoking, being overweight, inadequate exercise and a low intake of fruit and vegetables [5]. These lifestyle risk factors are more common in people with schizophrenia than in the general population [6–9].

In a North-American review, 42% of individuals with schizophrenia were reported to be obese [body mass index (BMI) ≥27 kg/m^2^] compared with 27% of the general population [6]. McCreadie and colleagues [7, 8] showed that the diets of people with schizophrenia in Scotland were less healthy than those of the general population on a range of parameters. Short-term efforts to improve diet in individuals with schizophrenia have been shown to be of only limited benefit [10] with the implication that any intervention must be long-term to be effective. Studies have repeatedly reported high rates of smoking in those with schizophrenia [8]. Information about exercise levels in schizophrenia is scanty, but clinical experience suggests it is often poor.

The high prevalence of poor diet, inadequate exercise and obesity in schizophrenia may partly reflect the associated socio-economic disadvantages of the illness, and many sufferers are unable to gain paid employment. In addition, core psychiatric symptoms including avolition and tiredness may contribute. Antipsychotic medication can cause metabolic derangements, including hyperglycemia and hyperlipidaemia [11, 12] as well as weight gain [12, 13] which if sustained can contribute to CVD. Other psychiatric medications, including mood stabilisers and some antidepressants, can also cause weight gain [2]. In summary, the excess of CVD in schizophrenia appears multifactorial.

Given the evidence of suboptimal lifestyle choices in people with SMI, the aims of this study were to determine the pattern of dietary intake and exercise in a representative group of individuals with schizophrenia in a UK inner city area and to determine whether age predicted the presence of metabolic syndrome in this group.

## Methods

The study was carried out at two Community Mental Health Centres in Salford, an inner city area in North West England, UK. The study was approved by the local Salford Ethics Committee and the Trust Research and Development Department. All outpatients aged between 16 and 65 years of age who were prescribed a neuroleptic drug and had a diagnosis of schizophrenia or schizoaffective disorder were eligible to enter the study. A series of consecutive outpatients were asked to consider entering the study. The majority of participants (23 out of 37) were living alone.

With regard to the characteristics of responders vs non-responders, in relation to clinical variables, for those non-responders for whom data are available (21 service users), there was no significant difference in age, BMI, blood glucose, and cholesterol level between the groups.

Patients who consented attended for a single assessment in a fasted state (i.e. not having had anything to eat since 22.00 h the night before). They completed a short interview to assess diet and activity in the previous week (Additional file 1). Assessment of diet and exercise was based on validated assessment tools [14]. Activity was rated as vigorous or moderate using the definitions given in Additional file 1. Subjects also completed Likert scales (rated 1–10) that assessed their views of diet, activity and medication compliance.

Each participant underwent basic anthropometric measurements, namely height, weight, and waist: hip ratio. Pulse and blood pressure were checked using a validated semi-automatic Omron HEM-705CP monitor (Omron Healthcare, Kyoto, Japan). The interviews and anthropometric measurements were undertaken by one of two trained research nurses. Socio-demographic details, details of psychiatric and medical history, and current prescribed medication were taken from the medical notes.

A fasting blood sample was taken for a variety of biochemical tests including serum glucose, lipids and prolactin. Apart from prolactin measurement, all assays were performed on the Roche Modular System (Burgess Hill, West Sussex, UK). Prolactin was assayed on the Siemens Immulite 2000 automated analyser (Siemens Healthcare Diagnostics, Frimley, Camberley, Surrey, UK).

### Metabolic syndrome definition

According to the 2005 IDF definition [15], for a person to be defined as having the metabolic syndrome they must have the following:

Central obesity (defined as waist circumference ≥94 cm for European men and ≥80 cm for European women, with ethnicity specific values for other groups, specifically 90 cm for South Asian and Oriental origin men) plus any two of the following four factors (all but two of the participants in this study were of European origin):

- raised TG level: ≥150 mg/dL (1.7 mmol/L), or specific treatment for this lipid abnormality
- reduced HDL cholesterol: <40 mg/dL (1.03 mmol/L) in males and <50 mg/dL (1.29 mmol/L) in females, or specific treatment for this lipid abnormality
- raised blood pressure: systolic BP ≥ 130 or diastolic BP ≥ 85 mmHg, or treatment of previously diagnosed hypertension
- raised fasting plasma glucose (FPG) ≥100 mg/dL (5.6 mmol/L), or previously diagnosed type 2 diabetes if >5.6 mmol/L or 100 mg/dL.

## Results

### Sample characteristics

The response rate among those eligible to enter the study was 41%. Twenty-five men and twelve women participated in the study. All had schizophrenia or schizoaffective disorder.

The mean duration of illness was 11.6 years (95% confidence interval: 7.3–18.2). The mean age was 46.2 years (46.2–49.2). Of the group, 30 (81%) were unemployed, four were in paid employment, one was in voluntary employment, one was retired, and one was off work due to sickness at the time of interview. The majority of participants (35 out of 37) were of White European origin, with one of South Asian ethnicity, and one of Chinese ethnicity. In keeping with the protocol, all patients were taking neuroleptic medication. The key results are summarised in Table 1.

**Table 1:** Details of sample and key results

Socio-demographic factors	Number and % of patients (unless otherwise specified)
Mean age (95% CI), yrs	46.2 (43.2–49.2)
Male (%)	25 (67.6)
Mean duration of illness (95% CI), yrs	11.6 (7.3–18.2)
Caucasian (%)	35 (94.6)
*Lifestyle parameters*	
5 portions or more of fruit and vegetables per day (%)	13.5
Fresh fruit at least once a week (%)	62.5
Vigorous exercise taken once a week for ≥10 min (%)	29
BMI ≥30 (%)	47.2
Current smoker (%)	64.9
Weekly alcohol > safe limits (%)	15
*Blood parameters*	
Fasting glucose elevated >6.0 mmol/L (%)	21.2
Cholesterol >5 mmol/L (%)	48
Prolactin elevated >1000 mu/L (%)	16.7
*Miscellaneous*	
Blood pressure >140/90 mmHg (%)	32.4
Metabolic syndrome (%)	67.6
Regard diet as unhealthy (%)	54.1
Regard themselves as physically inactive (%)	51.4

Of the participants, 20 out of 37 were taking oral atypical agents (eight were taking Clozapine) with 13 on depot neuroleptics (of which two were receiving depot Risperidone) and four on mood stabilisers as the primary psychotropic agent.

### Smoking and alcohol use

Twenty-four out of 37 subjects (64.9%) were current smokers, six (16.2%) were ex-smokers with seven (18.9%) never having smoked. Of the current smokers, four smoked between 1 and 10 cigarettes per day, nine between 11 and 20 cigarettes per day, and eleven between 21 and 60 cigarettes per day. 15% of male subjects consumed above the recommended safe levels of alcohol in a week (21 units per week). No women consumed above the recommended safe levels of alcohol in a week (14 units per week). Of the total sample of 37 patients, 14 took no alcohol in a week.

### Weight and related measurements

Within the group, BMI ranged from 18.4 to 52.4 kg/m^2^ (normal range: 18.5–25.0 kg/m^2^; World Health Organisation [16]). Mean (95% CI) BMI for men was 31.2 (28.2–34.3) kg/m^2^, and for women was 31.8 (26.3–37.3) kg/m^2^. 47.2% of the group had a BMI in the obese range (≥30.0 kg/m^2^). Thirty-two (86.5%) reported that they did not find it difficult to put on weight.

**Fig. 1:**
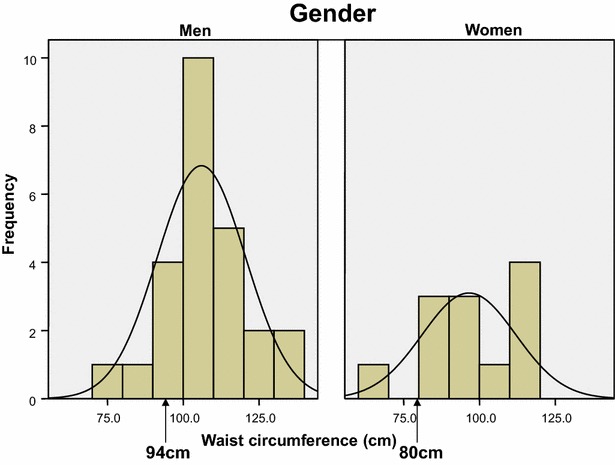
Distribution of waist circumference for men and women. The 80 and 94-cm marks on the figure indicate the cut points for the International Diabetes Federation [15] definition of central adiposity

For men (all of White European origin), mean (95% CI) waist circumference was 106.9 (100.8–112.9) cm (Fig. 1). For Caucasian men, central adiposity is defined as waist ≥94 cm [15]. For men (one was South Asian), waist circumference was 96.5 (86.7–106.3) cm (Fig. 1). For women of European and South Asian ethnicity, central adiposity is defined as waist ≥80 cm [16]. 92.0% of men had central adiposity as defined, as did 91.7% of women.

### Pulse and blood pressure

Mean (±SD) systolic blood pressure was 126 ± 19 mmHg and mean diastolic blood pressure was 80 ± 13 mmHg. 21.6% had a systolic blood pressure >140 mmHg and 45.9% a diastolic blood pressure >80 mmHg.

### Laboratory results

Four out of 37 patients were known to have diabetes, and three of the 37 patients (8.1%) had a fasting glucose between 6.1 and 6.9 mmol/L [17]. Fasting total cholesterol was >5 mmol/L in 48% of individuals with fasting LDL-cholesterol >3 mmol/L in 43.3% of patients. 16.7% of the patients had a serum prolactin >1000 μL, the threshold agreed by local endocrine services as meriting further investigation.

### Metabolic syndrome

Twenty-five patients (67.6%) would be categorised as having the metabolic syndrome using the International Diabetes Federation (IDF) Criteria [15]. This was more likely if the individual was older (odds ratio 1.4 (95% CI 1.32–1.48).

### Diet

Thirty-two participants completed the dietary questionnaire. A total of 13.5% of participants ate ≥5 portions of fruit and vegetables per day. Mean (±SD) portions of fruit per day were 1.1 ± 1.0 and of vegetables were 1.7 ± 1.2. Total fruit and vegetable portions were 2.8 ± 1.8. For the group, oily fish was eaten on average on 0.5 ± 0.6 days of each week.

Fruit was only eaten on ≥3 days each week by 34.4% of the group, with 37.5% reporting not eating fruit on any day of the week (Table 2). Vegetables were eaten on ≥3 days of each week by 59.4% of individuals.

**Table 2:** Breakdown of fruit and vegetable intake in a week for the whole group

Number of days eating vegetables	% of group	Number of days eating fruit	% of group
0	12.5	0	37.5
1–2	28.1	1–2	28.1
3–5	34.4	3–5	18.8
>5	25.0	>5	15.6

With regard to takeaway foods, twelve (37.5%) did not have any in the previous week, 18 (56.3%) had 1–2 takeaways and two (6.3%) had >2 takeaways. For ready meals, the breakdown was similar with 50% (16 out of 32) having none, 34.4% (11/32) having 1–2 ready meals, 9.4% (3 out of 32) having 3–4 ready meals but 6.3% (2 out of 32) having ≥7.

For crisps, 18 out of 32 (56.3%) had no crisps in the last week and for bread 56.3% (18 out of 32) had white bread, 13 wholemeal/granary bread/brown bread, and one had no bread.

### Activity

**Fig. 2:**
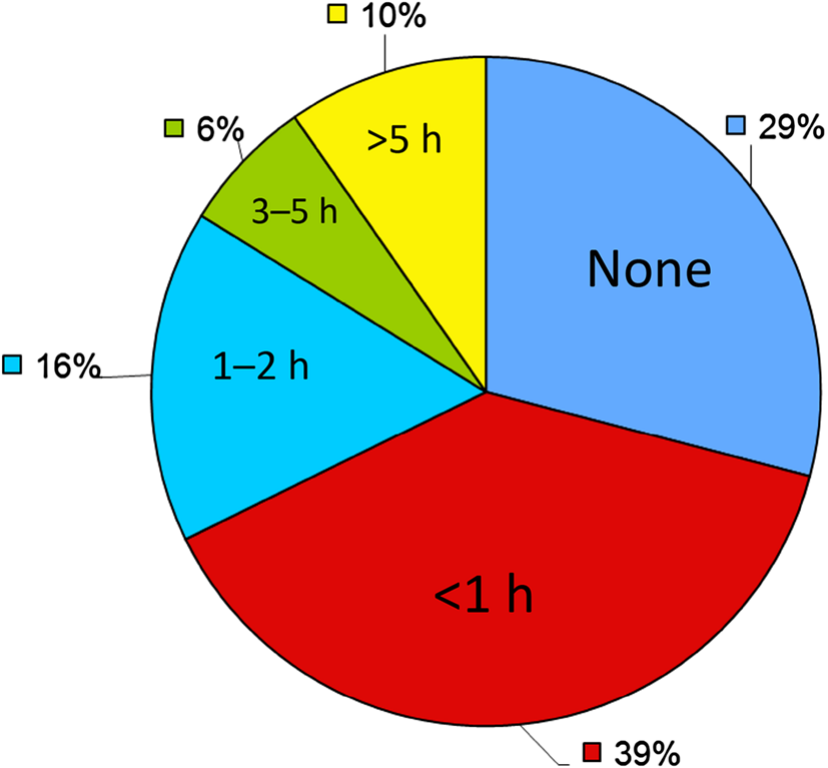
Moderate exercise activities (hours per week) in a typical week

41.9% of participants reported doing no vigorous activity in the last week (see Additional file 1 for definition). 35.5% did <1 h of vigorous activity per week. Only 29% did ≥10 min of vigorous activity per week. 29% described doing no moderate activity in any week and 39% of patients did so for <1 h per week (Fig. 2). Of 31 responders, 21 walked for <1 h each day, 14 said that they walked for ≥10 min each day with five walking once or not at all in any week, and the remaining twelve walking 2–6 times each week for >10 min.

### Likert scale ratings

The people studied displayed an understanding that their lifestyle was less healthy than might be achieved, in terms of both diet and exercise.

#### Diet

Patient ratings on a visual scale of 1–10 for the question ‘Would you describe your diet as healthy (average over the last 3 months)?’ gave a mean ± SD of 5.7 ± 2.2. The anchor points were 0 = very unhealthy, 10 = very healthy.

#### Activity

Patient ratings on a visual scale of 1–10 for the question ‘Would you consider yourself fairly physically active (average over the last 3 months)?’ gave a mean ± SD of 5.0 ± 2.9. The anchor points were 0 = very physically inactive, 10 = very physically active.

#### Medication compliance

Patient ratings on a visual scale of 1–10 for the question ‘Are you compliant with medication prescribed for you to take (over the last 3 months)?’ gave a mean ± SD of 9.8 ± 0.8. The anchor points were 0 = not compliant, 10 = very compliant.

If a score of <5.0 on the Likert scales is used to indicate that a patient does not think they have a healthy diet, and a score <5.0 as a patient regarding themselves as not physically active, then the percentage of patients who accepted that they had a problem in these two areas was 54.1 and 51.4%, respectively.

## Discussion

We report three important findings from this study: (i) a high prevalence of metabolic syndrome; (ii) a high prevalence of poor diet, smoking and inadequate exercise; and (iii) that a high proportion of patients have insight into their unhealthy lifestyles. In relation to the third finding, we believe that an insight into lifestyle has not previously been reported. The fact that our subjects showed some degree of insight into their lifestyle problems indicates that there is potential for working with this group to improve the quality of diet and to increase the amount of exercise taken each day [10, 18].

In terms of methodology, the study sample was small but it is representative of patients with schizophrenia and schizoaffective disorder. In the current study, we approached a consecutive series of outpatients and just over 40% took part in the study. This is comparable with contemporary analysis of rates of screening in UK Primary Care [19].

If there is a sampling bias, it is likely that more motivated patients with healthier lifestyles took part. Patients were asked to attend in a fasted state and this was checked with them on the day that they attended. All anthropometric measurements were taken by one of two trained research nurses ensuring accuracy and consistency. The cut-offs that were used to define insight into lifestyle on the Likert scales have not been formally assessed; however, assessment of diet and exercise was based on validated assessment tools [14].

The prevalence of metabolic syndrome in our group was 67.6%. This is a major concern because metabolic syndrome is a strong predictor of CVD [15, 19, 20] and of cardiovascular death [21]. The rates of metabolic syndrome are similar to other studies. In terms of components of the metabolic syndrome, most patients had a BMI and waist circumference above normal thresholds, with 92% of both men and women having central adiposity. Perhaps surprisingly, only a fifth of patients had an elevated systolic blood pressure.

Many individuals did not follow the recommendations for dietary intake of fruit and vegetables [22]. A significant proportion did not exercise as much as is recommended [17]. Levels of smoking were high. These results mirrored the seminal work done on lifestyle in schizophrenia conducted by McCreadie et al. [7] in the 1990s and subsequently [8, 9]. It is of concern that there has been no improvement in the intervening period. In particular, the last decade has seen investment in strategies to reduce smoking in the general population which have been successful. In contrast, smoking levels remain high in those with SMI. Our data show that the problems noted by McCreadie [8] in a Scottish sample, namely high levels of smoking, poor diet and low levels of exercise are also seen in England, increasing the likelihood that these findings represent the national picture.

Regular health screening, including measurement of fasting lipids and glucose, is recommended for those with SMI and those treated with antipsychotics in many treatment guidelines [23]. In addition, it is generally agreed that lifestyle interventions are necessary to improve physical health of SMI patients [24, 25]. In non-psychiatric patients at high risk of developing diabetes, simple lifestyle adjustments have been shown to reduce diabetes risk. These interventions include decreasing calorific and fat content of food and increase fibre intake, increasing intake of fruit and vegetables, eating complex rather than simple carbohydrates (e.g. whole-wheat bread rather than refined white bread) and avoiding sugary drinks, as well as carrying out exercise for at least 10–15 min per day. Advice to the general population to improve fruit and vegetable intake has been shown to be successful, at least in the short term [26].

In an intervention study [9], the diet of people with schizophrenia improved when they were given free fruit and vegetables; however, this was not sustained after withdrawal of the intervention, although there was a trend for the return to pre-intervention consumption to be more gradual when the free food intervention was combined with dietary advice.

A significant proportion of patients in our study had hyperprolactinaemia. This is a recognised adverse effect of many antipsychotics [27, 28]. The propensity to cause hyperprolactinaemia varies significantly between different atypical antipsychotics. Raised prolactin can be asymptomatic, leading to various acute adverse effects, and can also result in long-term medical problems, including osteoporosis.

We do not have a direct comparison group from the general population. However, a detailed survey of the Salford UK population in terms of lifestyle is underway (www.citizenscientist.org.uk). The majority of the participants were living alone, so collection from someone cohabiting with them was not possible.

It could be argued that the quality of diet found in our study group is not significantly different from that of people in a similar socio-demographic situation [29]. However, the high rates of diabetes and cardiovascular events [2–4] in SMI define them as a high-risk group for cardiometabolic disease and, as such, there is the potential for targeted intervention to produce benefit.

We recommend the following principles of management to reduce cardiometabolic risk:

- Potential pre-diabetes states should be investigated and managed as per agreed guidelines for the general population but with annual screening for this recommended for those with psychosis receiving antipsychotic medications. The prescription of metformin for those not responding to or not adherent to intensive lifestyle interventions needs to be considered in the context of the individual service user.
- Diabetes should be managed by the family practitioner or a specialist physician where necessary.
- Dyslipidemia, especially in the context of a patient with diabetes, should be actively managed according to existing guidelines for the general population. There is no contra-indication to the prescription of a statin.
- Hypertension should be managed according to national guidelines. There is no contra-indication to prescription of antihypertensive medications.
- Smoking is an important additive risk factor for diabetes and cardiovascular disease and service users who smoke should be referred to smoking cessation services.

Our data show that there is scope to achieve a more healthy lifestyle in individuals with SMI. Such improvements require collaboration between local Mental Health providers and General practitioners plus specialist services, particularly dietetics, community health trainers and occupational therapy. It is likely that in many areas of the UK, and elsewhere, significant healthcare system re-design will be necessary to achieve this goal [30].

One of the founding principles of the NHS in 1948 was that it should strive to improve the health of the individual and the population and prevent disease. The needs of the service users lie at the heart of this fundamental mission and of other healthcare providers across the world responsible for the welfare of patients with schizophrenia and other forms of severe enduring illness.

## Conclusions

Within this sample, there was a high prevalence of poor diet, smoking and inadequate exercise. Many did not follow national recommendations for dietary intake of fruit and vegetables and daily exercise. These factors probably contribute to the high prevalence of metabolic syndrome. Many had insight into their unhealthy lifestyles. Thus, there is potential for interventions to improve lifestyle factors and reduce the risk of cardiometabolic disease.

## Additional file

**Additional file 1.** Comparison of absorption of dietary macronutrients and biochemical markers related to obesity in a population at the extremes of weight distribution.

## Abbreviations

BMI: body mass index
CVD: cardiovascular disease
FBG: fasting blood glucose
IDF: International Diabetes Federation
SMI: severe and enduring mental illnesses
