# Supplementary material for: Lifestyle factors and the metabolic syndrome in Schizophrenia: a cross-sectional study
Source: Ann Gen Psychiatry. 2017 Feb 15;16:12. doi: 10.1186/s12991-017-0134-6 (PMC5310063; doi:10.1186/s12991-017-0134-6)
Supplement: Supplementary file 1 — Additional file 1. Comparison of absorption of dietary macronutrients and biochemical markers related to obesity in a population at the extremes of weight distribution. [file 12991_2017_134_MOESM1_ESM.doc]

# Appendix 1 (version 1; May 5, 2006)

# Comparison of absorption of dietary macronutrients and biochemical markers related to obesity in a population at the extremes of weight distribution

# NAME DOB DATE

#

# Last 7 days dietary questionnaire

# I am going to ask you a few questions about your diet. I would like you to think about your food and drink over the past 7 days or during a typical week.

# 1a. On how many days did you eat fresh fruit including tinned or frozen?

# None

# 1-2

# 3-5

# more than 5

# b. On average how many portions of fruit did you have in a day (1 portion is 1 apple, 1 banana, 1 orange, 15 grapes or strawberries, 2 satsumas etc)?

# 2a. On how many days did you eat salad or vegetables excluding potatoes? This includes fresh, frozen and tinned vegetables, and fresh vegetable soups.

# None

# 1-2

# 3-5

# More than 5

# b. On average how many serving spoons/portions of vegetables/salad do you eat a day?

# 3a. On how many days did you have pulses (this includes all types of beans, including baked beans, and lentils)

# None

# 1-2

# 3-5

# More than 5

# b. On average how many serving spoons of pulses do you eat a day?

# 4a. How many times in the week did you have take-aways – eg MacDonalds, Pizza Hut, fish and chips, Chinese, Indian etc, or eat out in restaurants?

# None

# 1-2

# 3-4

# 5-6

# 7 or more

# 5. How many times in the week did you have ready meals/convenience foods/microwave meals? (This includes freezer items that are eaten immediately after warming and pies/fish in various coatings.)

# None

# 1-2

# 3-4

# 5-6

# 7 or more

# 6. On how many days did you cook fried foods at home (other than those mentioned above)? This therefore includes fried meats (eg sausages, bacon) chips (including oven/microwave chips), vegetables (eg fried mushrooms, tomatoes), etc. [not stir-fries]

# None

# 1-2

# 3-5

# More than 5

# 7. On how many days did you eat chicken, or turkey?

# None

# 1-2

# 3-5

# More than 5

# 8. On how many days did you eat oily fish: eg salmon, trout, mackerel, sardines, tuna?

# None

# 1-2

# 3-5

# More than 5

# 9. On how many days did you eat white fish cooked at home eg cod, haddock, sole, hake – can be baked, boiled or fried?

# None

# 1-2

# 3-5

# More than 5

# 10. On how many days did you eat meat or dishes containing meat eg Shepherds pie, lasagne etc

# None

# 1-2

# 3-5

# More than 5

# 11. How many eggs did you eat in the week?

# None

# 1-2

# 3-4

# 5-6

# 7 or more

# b. Do you mostly have your eggs boiled, poached, as omelette, scrambled eggs or fried?

# 12. What type of spread do you use on bread? Is this Low, very low or normal fat?

# None

# NAME of spread

# 13. What kind of milk do you use?

# None

# Skimmed

# Semi-skimmed

# Full fat

# Other

# b. If YES to full fat, how much per day? (eg 1 pint, half pint etc)

# 14. How many times in the week do you eat cheese or cook with cheese (eg cheese sauce)

# None

# 1-2

# 3-4

# 5-6 What type of cheese do you eat?

# 7 or more

# 15. How many times in the week did you have sugary snacks between meals eg chocolate, cakes, sweets, sweet biscuits (ie not crackers, digestives etc)?

# None

# 1-2

# 3-4

# 5-6

# 7or more

# 16. How many days did you have sweet desserts after meals eg ice cream, pies etc?

# None

# 1-2

# 3-4

# 5-6

# 7 or more

#

# 17. How many times in the week did you have drinks containing sugar – this includes tea or coffee with sugar added, fizzy sugary drinks eg coke, pepsi, or fruit juices (exclude cordials with no added sugar)

# None

# 1-2

# 3-4

# 5-6

# 7 or more

# 18. How many bags of crisps did you have in the last week (small bags or equivalent)?

# None

# 1-2

# 3-4

# 5-6

# 7or more

# 19. How many times in the week did you eat a handful of any nuts?

# None

# 1-2

# 3-5

# 6-8

# More than 9

# 20. Which cereal do you usually have for breakfast?

# None

# NAME of cereal

# 21. What type of bread do you usually eat?

# White

# Brown

# Wholemeal

# Granary

# 22. How many units of the following types of alcohol did you drink in the week?

# None

# Beer/lager

# Red wine

# White wine

# Spirits

# (Operator to explain that 1 unit of wine = 1 standard wine glass, 1 unit of beer/lager = half a pint, and 1 unit of spirits = 1 pub measure)

**Last 7 days physical activity questionnaire**

I am now going to ask you about the time that you spent being physically active in the last 7 days or in a typical week.

1a. During the last 7 days or in a typical week, on how many times did you do vigorous activities for at least 10 min or more?

These activities make you breathe much harder than normal and may include heavy lifting, digging, aerobics, fast bicycling, running, fast swimming etc.

- 1. None
  2. 1-2 times in a week
  3. 3-5 times in a week
  4. > 5 times in a week

**(For the operator: If the answer to above question is a), then please move to question 2a)**

1b. During the last 7 days or in a typical week how many hours in total did you spend doing these vigorous activities

1. <1 hr in a week
2. 1-2 hrs in a week
3. 3-5 hrs in a week
4. >5 hrs in a week

2a. During the last 7 days or in a typical week how many times did you do moderate physical activity?

These activities make you breathe somewhat harder than normal and may include carrying light loads, bicycling at regular pace, gentle swimming, hill walking, vacuuming and others. Again think about activities that you did for at least 10 min.

1. None
2. 1-2 times in a week
3. 3-5 times in a week
4. more then 5 times in a week

**(For the operator: if the answer to question 2 is a), then please move to question 3a.)**

2b. How much time do you think you spent doing those activities in last 7 days or a typical week?

- - 1. Up to 1 hour
    2. 1 –2 hours in a week
    3. 3-5 hours in a week
    4. more then 5 hours in a week

3a. During the last 7 days or in a typical week how many times did you walk continuously for at least 10 min or more?

This includes at work and at home, walking to travel from place to place and any other walking for exercise, or leisure.

1. Every day
2. 4-6 time in a week
3. 2-3 times in a week
4. 0-1 time in a week

3b. What is the total amount of time you spent walking each day? Please tell us about a typical day. If your activities vary from day to day then please tell us about last Wednesday.

1. Up to 1 hour in a day
2. 1-2 hours in a day
3. 3-5 hours in a day
4. more then 5 hours a day

4. During the last 7 days how much time did you spend **sitting or lying** on a weekday?

This includes time spent at work, at home, visiting friends, sitting or lying on sofa, watching television etc., **this does not include sleeping**.

(If the pattern of the time spent varies widely then take a typical day or last Wednesday)

1. 1-2 hours per day
2. 3-5 hours per day
3. 6-9 hours per day
4. more then 9 hours a day
